# Measurement of area difference ratio of Photoplethysmographic pulse wave in patients with pre-eclampsia

**DOI:** 10.1186/s12884-018-1914-y

**Published:** 2018-07-03

**Authors:** Ying Feng, Dan Drzymalski, Baihui Zhao, Xuan Wang, Xinzhong Chen

**Affiliations:** 1 Department of Anaesthesia, Women’s Hospital, School of Medicine, Zhejiang University, Xueshi Rd 1, Hangzhou, 310006 China; 2 Tufts Medical Center, Tufts University School of Medicine, 800 Washington Street, Boston, MA 02111 USA; 3 Department of Obstetrics, Women’s Hospital, School of Medicine, Zhejiang University, Xueshi Rd 1, Hangzhou, 310006 China; 4 School of Medical Instruments, Shanghai University of Medicine & Health Sciences, 257 TianXiong Rd, Pudong, ShangHai, 201318 China; 5 Department of Anaesthesia, Women’s Hospital, School of Medicine, Zhejiang University, Xueshi Rd 1, Hangzhou, 310006 China

**Keywords:** Photoplethysmography, Area difference ratio, Hypertension, Preeclampsia

## Abstract

**Background:**

Preeclampsia (PE) is associated with an increase in maternal arterial stiffness, which may be reflected by photoplethysmography (PPG) of the pulse wave. The aim of this study was to investigate area difference ratio (ADR), a novel parameter derived from PPG, in women with and without preeclampsia.

**Methods:**

Patients with and without preeclampsia in the third trimester were enrolled. All patients had photoplethysmography of the pulse wave assessed. ADR was compared between the two groups.

**Results:**

Seventy-two patients in the third trimester of gestation, of which 36 had preeclampsia and 36 did not, were enrolled. The ADR was lower in the preeclampsia group vs. the non-preeclampsia group (0.725 [IQR 0.681–0.779] vs. 0.752 [IQR 0.717–0.910], *P* < 0.01).

**Conclusions:**

Measuring the ADR through analyzing PPG of the pulse wave may be a useful diagnostic tool in patients with preeclampsia.

## Background

Preeclampsia remains one of the leading causes of maternal and fetal morbidity and mortality worldwide, affecting 2–3% of all pregnancies in the USA and 7.5% globally [1, 2]. The disease is characterized by hypertension, proteinuria and vascular dysfunction [3–5], and the pathophysiology of preeclampsia may involve an increase in arterial wall rigidity as a result of maternal endothelial dysfunction [6, 7]. Arterial stiffness can be assessed by measuring various parameters, including pulse wave velocity (PWV) and augmentation index (AIx) [6, 8–12]. As arterial stiffness is associated with an increased risk of having a cardiovascular event in healthy non-pregnant subjects [13], PWV and Aix may be helpful in predicting morbidity and mortality in preeclampsia. Similarly, PWV and Aix may also be used to provide information on arterial compliance in preeclampsia [8, 11, 14, 15].

Photoplethysmography (PPG) is a non-invasive and readily available optical technique that uses infrared light to illuminate the fingertip tissue and measures variations in light intensity that correspond to blood vessel volume [16, 17]. Previous studies have demonstrated that the contour of the PPG contains similar information to that of the peripheral pressure wave and may be used to evaluate arterial stiffness [18–20].

The diastolic decay constant (a function of vessel resistance and compliance) [21, 22] is associated with physiological changes of the cardiovascular system and is useful in vascular assessment [20, 22–25]. However, it is difficult to extract from the finger PPG pulse waveform. As such, the area difference ratio (ADR) was developed using a novel, non-iterative, shape method from the PPG waveform to serve as a parameter that is more readily established than the diastolic decay constant.

The aim of this study was to better understand the pathophysiology of preeclampsia by measuring the ADR in patients with or without preeclampsia at the time of disease. Our hypothesis was that the ADR would be lower in patients with preeclampsia.

## Methods

### Study population

The study protocol was approved by the Research Ethics Committee of the Women’s Hospital, School of Medicine, Zhejiang University, and written informed consent was obtained from all patients who participated in the study. Patients with singleton pregnancies of at least 28 weeks gestational age were enrolled during a routine prenatal screening at the obstetrics clinic into one of two groups: the preeclampsia group (PE) or the non-preeclampsia group (Non-PE). Patients were enrolled in the PE group if they had a systolic blood pressure (SBP) ≥140 mmHg or diastolic blood pressure (DBP) ≥90 mmHg measured on two separate occasions at least 6 h apart, and proteinuria of ≥0.3 g/L in a 24 h urine collection that started after 20 weeks of pregnancy [26]. Pregnant women were excluded if they had gestational diabetes, essential hypertension, renal disease, history of tobacco use, and alcohol or illicit drug abuse.

### Pulse wave analysis

**Fig. 1:**
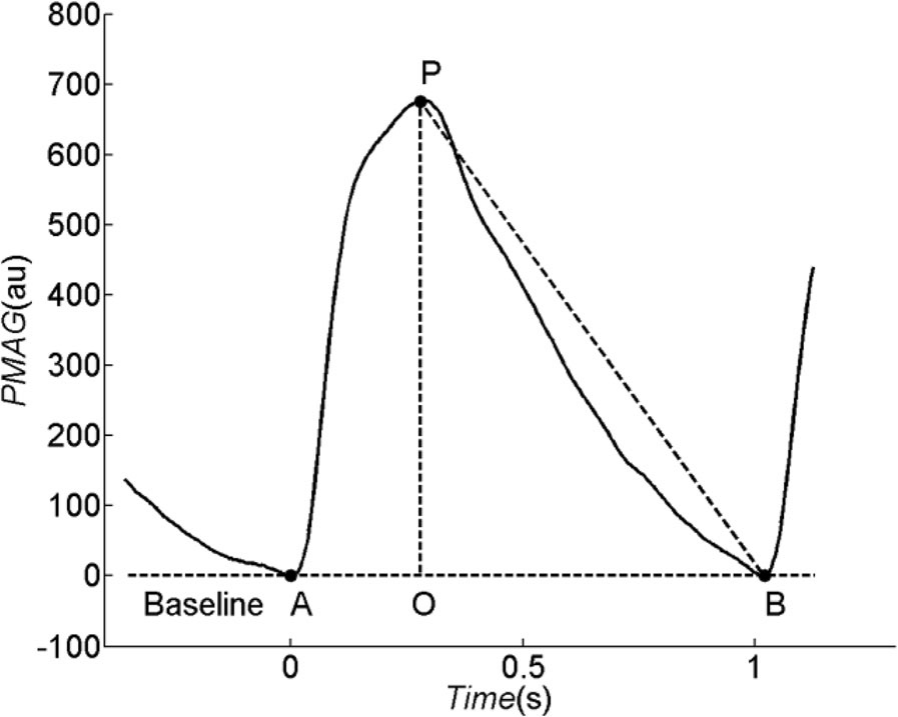
A representive beat of pulse photoplethysmographic waveform. Baseline is corrected to 0. Point A, P and B are the onset, peak and end position of pulse, respectively. Point O is the vertical projection of point P on the baseline. PPG signal (−) is shown with PPG magnitude in arbitrary units (au)

Women who consented to the study were instructed to fast for at least 12 h prior to the study. Baseline demographic data were gathered upon the start of the study. All participants were instructed to sit comfortably with her arm supported on a table in a room whose temperature was maintained at 24 °C. After at least 10 min of rest, the SBP and DBP were measured twice and the mean calculated. An adult oxygen sensor (DS-100A Durasensor, OxiMax, Nellcor Puritan Bennett, Inc.) was placed on the index finger of the non-dominant arm of pregnant woman to detect and collect PPG signal at a rate of 250 Hz. PPG measurements were performed 3 times over 5 min intervals and the mean calculated. Data was stored on a computer with specialized software we developed to calculate ADR, PPG amplitude (PPGA) and pulse beat interval (PBI). ADR was calculated as the difference between the area of the triangle (St) formed by points B, P, and O, and the area under the curve of the pulse (Sp) but above the horizontal line formed by points B and O, divided by St, as follows (see Fig. 1 for points referenced) [21]: ADR = (St – Sp) / St. PPGA was calculated as the difference of magnitude between point P and baseline. PBI was calculated as the time difference between point A and point B.

### Statistical analysis

All statistical analyses were performed using Graphpad Prism 4 (Graphpad software Inc., San Diego, CA, USA). A *P*-value of < 0.05 was considered statistically significant. The Kolmogorov-Smirnov test was used to assess for Gaussian distribution. Data were expressed as mean ± standard deviation (S.D.) or as median and interquartile range (IQR) for normally and non-normally distributed data, respectively. Comparisons between groups were performed using Student’s t-test and the Chi-squared test were used for parametrically distributed continuous and categorical variables, respectively, and the Mann-Whitney U test was used for non-parametrically distributed continuous variables. A multivariate regression model was used to analyze the association of ADR with maternal demographic and hemodynamic data.

## Results

A total of 72 pregnant women, 36 in the PE group and 36 in the Non-PE group, enrolled in the study between June 1 and December 31, 2016. The PE group consisted of 30 patients with mild and 6 patients with severe preeclampsia. Twenty-three patients in the PE group were taking antihypertensive medications, including β-blockers (14 patients), β-blockers and Calcium channel blockers (9 patients), or Calcium channel blockers (3 patients). A total of 26 patients had early onset (< 34 weeks) preeclampsia and 10 had late onset (> 34 weeks) preeclampsia.

Baseline demographic data of study participants are presented in Table 1. There were no statistically significant differences between the groups in maternal age, gestational age at enrollment, or fetal sex. The PE group exhibited higher weight, higher body mass index, shorter height, and lower birth weight. Gestational age at delivery was significantly earlier in the PE group than in normal pregnant women.

**Table 1:** Demographic data for pregnant women: normal and preeclampsia

parameter	Non-PE (*n* = 36)	PE (*n* = 36)	*P* Value
Maternal age (years)	30.0 ± 3.6	31.3 ± 3.9	0.195
Gestational age at enrollment (weeks)	30.8 ± 2.2	31.5 ± 2.5	0.207
Gestational age at delivery (weeks)	39.1 ± 0.9	34.1 ± 3.2	< 0.0001
Maternal height (m)	1.63 ± 0.05	1.60 ± 0.05	0.045
Maternal weight (kg)	67.1 ± 9.4	73.3 ± 7.5	0.005
Body mass index (kg/m^2^)	25.4 ± 3.2	28.7 ± 2.3	< 0.001
Birth weight (g)	3514 ± 375	2283 ± 1010	< 0.0001
Fetal sex (male/female)	(16/20)	(19/17)	0.479

Values are given as mean ± S.D. or as median (IQR) for normally and non-normally distributed data, respectively

Multivariate regression analysis found no significant associations between ADR and height (*P* = 0.94), weight (*P =* 0.83), SBP (*P =* 0.85), and DBP,(*P =* 0.83).

**Fig. 2:**
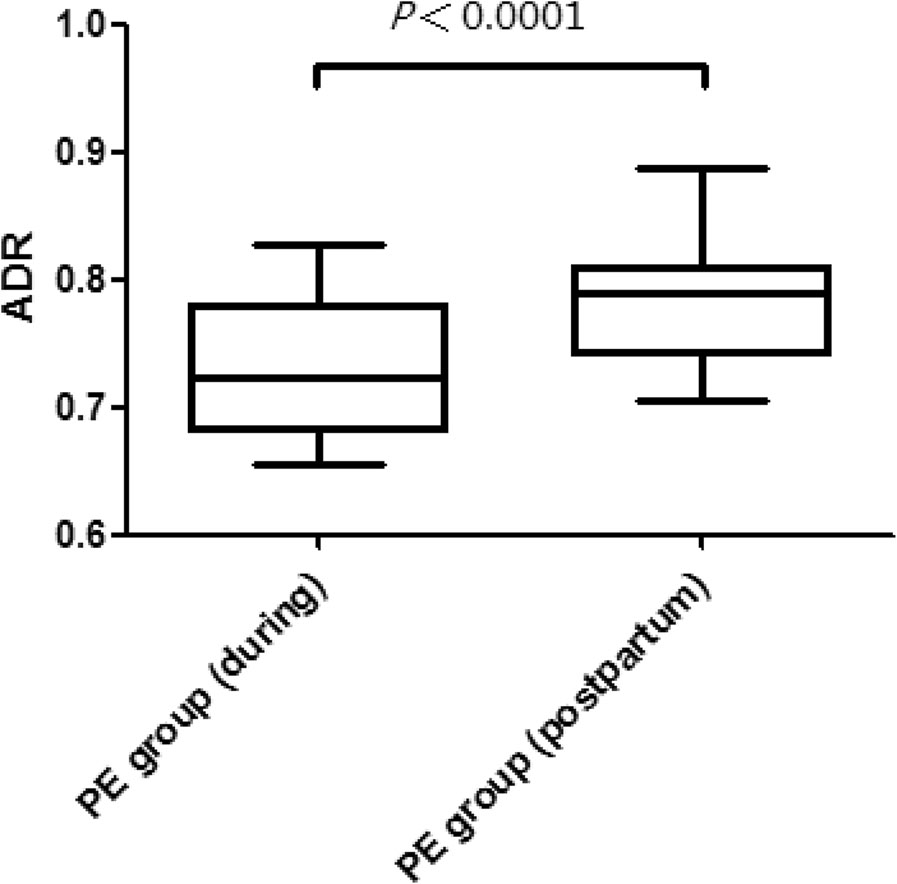
Box and whisker plots comparing the ADR in the PE group during pregnancy (left), and postpartum (right). Boxes represent IQR, where the line represents the median. Whiskers at top and bottom of the box represent the highest and lowest values

SBP, DBP, mean arterial pressure, PPGA, PBI, and ADR are presented in Table 2. The PPGA and PBI were not significantly different between the groups. Conversely, patients in the PE group had a lower ADR decreased and higher SBP, DBP, and MAP. The ADR returned to baseline 42 days postpartum in the PE group (see Fig. 2).

**Table 2:** Vascular characteristics of the study populations

parameter	Non-PE (*n* = 36)	PE (n = 36)	*P* Value
Heart rate (beats/min)	75.9 ± 9.7	76.4 ± 9.2	0.679
SBP (mmHg)	115.8 ± 9.2	152.6 ± 14.8	< 0.001
DBP (mmHg)	69.6 ± 7.6	99.3 ± 3.0	< 0.001
Mean arterial pressure (mmHg)	85.0 ± 7.5	117.1 ± 6.1	< 0.001
PPGA(au)	280.4 (215–372.4)	268.4 (158.6–428.1)	0.853
PBI(s)	0.628(0.543–0.651)	0.625 (0.608–0.699)	0.205
ADR	0.752 (0.717–0.910)	0.723 (0.681–0.779)	< 0.01
ADR (postpartum)	0.778 (0.723–0.813)	0.789 (0.742–0.810)	0.404

Values are presented as mean ± S.D. or as median (IQR) for normally and non-normally distributed data, respectively

## Discussion

In this study, we observed that patients with preeclampsia have a lower ADR compared to those without preeclampsia during the early third trimester. No differences were observed in PPGA and PBI between the two groups.

Our primary finding that preeclampsia was associated with a lower ADR is consistent with our initial hypothesis. The ADR is closely correlated to the diastolic decay constant, a parameter that describes the exponential rate at which arterial pressure decreases during diastole [21, 22]. An accelerated diastolic decay constant is associated with greater arterial stiffness and therefore hypertension. By measuring the diastolic decay constant with invasive arterial blood pressuring monitoring we can further characterize resistance and total arterial compliance [27]. In addition, one of the major advantages of the diastolic decay constant is that local factors, including perfusion, do not significantly alter its value [28–30]. However, the invasive nature of this test limits its clinical utility on the labor and delivery unit.

The major advantage of PPG is that it is a non-invasive technique that can measure various parameters associated with the pulse wave. While several parameters from the PPG pulse wave have been measured, including the PPG amplitude and the PPG notch position and notch relative amplitude, none have demonstrated clinical utility [31–34]. Furthermore, environmental factors, metabolic state, motion artifact and psychological wellbeing all influence these parameters and make interpretation of those parameters unreliable [35, 36]. On the other hand, the major advantage of the ADR is that the effect of PPG magnitude is eliminated in its calculation and therefore makes it more reliable [21]. The only limitation of the ADR is that pre-calibration is essential for a reliable measurement.

Preeclampsia is characterized by a marked increase in peripheral vascular resistance and vasoconstriction [3–5], and increased sympathetic vasoconstrictor activity has been demonstrated with measurements of muscle sympathetic nerve activity [3, 37]. The etiology of the increased sympathetic tone is likely endothelial dysfunction [38–41]. It is important to note that endothelial dysfunction may be present in preeclampsia as this is an important step in the development of atherosclerosis in patients with chronic hypertension [42]. In non-pregnant patients with chronic hypertension, vascular disease or diabetes, vascular stiffness may be due to the decline of vascular compliance [43–46].

It is important to note that our study has several limitations. First, given the study design (cross-sectional study of women with preeclampsia at 30 weeks gestational age), our results describe pathophysiological changes of preeclampsia but do not necessarily indicate that the ADR is a useful tool in the prediction of preeclampsia. Second, while we believe that ADR may be a useful measure of arterial stiffness, we did not correlate the ADR to other established measures of arterial stiffness (e.g. PWV, Aix). Future studies should attempt to make that correlation. Third, given that increased sympathetic activity may be characteristic of pregnancy in the absence of neurological disease, digital blood flow and thus the waveform may have been affected by peripheral vasoconstriction. Nevertheless, the diastolic decay constant is a value derived from the waveform and might not be as significantly influenced by local perfusion.

## Conclusions

Our study offer a new insight into the pathophysiologic features in preeclampsia. The results suggest that the ADR may be decreased in patients with preeclampsia, but our study does not tell us about the ability of the ADR to predict preeclampsia. Future studies should examine how ADR may be used to predict preeclampsia throughout pregnancy, whether predicting it early or later gestation.

## Abbreviations

ADR: area difference ratio
Aix: augmentation index
AS: arterial stiffness
DBP: distolic blood pressure
MBP: mean blood pressure
PBI: pulse beat interval
PE: preeclampsia
PPG: photoplethysmography
PPGA: photoplethysmography amplitude
PWV: pulse wave velocity
SBP: systolic blood pressure
